# Discovery of intramolecular *trans*-sialidases in human gut microbiota suggests novel mechanisms of mucosal adaptation

**DOI:** 10.1038/ncomms8624

**Published:** 2015-07-08

**Authors:** Louise E. Tailford, C. David Owen, John Walshaw, Emmanuelle H. Crost, Jemma Hardy-Goddard, Gwenaelle Le Gall, Willem M. de Vos, Garry L. Taylor, Nathalie Juge

**Affiliations:** 1 The Gut Health and Food Safety Institute Strategic Programme, Institute of Food Research, Norwich Research Park, Norwich NR4 7UA, UK; 2 Biomolecular Sciences Building, University of St Andrews, St Andrews KY16 9ST, UK; 3 School of Computing Sciences, University of East Anglia, Norwich NR4 7TJ, UK; 4 Laboratory of Microbiology, Wageningen UR, Building 316, Dreijenplein 10, 6703 HB Wageningen, The Netherlands; 5 Departments of Bacteriology and Immunology and Veterinary Bioscience, University of Helsinki, PO Box 66, FI-00014 Helsinki, Finland

## Abstract

The gastrointestinal mucus layer is colonized by a dense community of microbes catabolizing dietary and host carbohydrates during their expansion in the gut. Alterations in mucosal carbohydrate availability impact on the composition of microbial species. *Ruminococcus gnavus* is a commensal anaerobe present in the gastrointestinal tract of >90% of humans and overrepresented in inflammatory bowel diseases (IBD). Using a combination of genomics, enzymology and crystallography, we show that the mucin-degrader *R. gnavus* ATCC 29149 strain produces an intramolecular *trans*-sialidase (IT-sialidase) that cleaves off terminal α2-3-linked sialic acid from glycoproteins, releasing 2,7-anhydro-Neu5Ac instead of sialic acid. Evidence of IT-sialidases in human metagenomes indicates that this enzyme occurs in healthy subjects but is more prevalent in IBD metagenomes. Our results uncover a previously unrecognized enzymatic activity in the gut microbiota, which may contribute to the adaptation of intestinal bacteria to the mucosal environment in health and disease.

## Abstract (web summary)

Mucosal sialoglycans contribute to host–microbe interactions at mucosal surfaces and impact bacterial colonization of the digestive system. Here the authors identify and characterize an intramolecular *trans*-sialidase produced by the gut bacterium *R. gnavus* ATCC 29149 that may contribute to adaptation to the mucosal environment.

## Graphical abstract

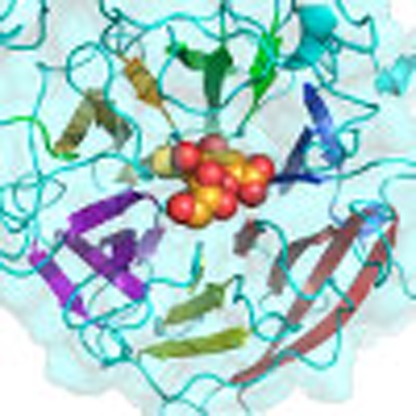

The human gut is populated with microorganisms that play important roles in health and disease^1^. The majority of the gut bacteria are believed to reside within the outer mucus layer covering the gastrointestinal tract epithelial cells^2^. Changes in the balance of different bacteria in our microbiota have been linked to inflammatory bowel diseases (IBD)^3^,^4^. There is an emerging paradigm that mucus is critical to maintain a homeostatic relationship with our gut microbiota, and that any deviation from this dynamic interaction has major implications for health (colitis, colorectal cancer, infections and so on)^5^,^6^,^7^,^8^. For example, patients suffering from IBD have a disproportionate representation of mucin degraders, such as *Ruminococcus gnavus*^9^, a common species of gut bacteria found in over 90% of people^10^.

Mucins are the most abundant protein in mucus, with a ‘bottle brush' appearance of sialic acid-capped O-glycan chains attached to the protein backbone via serine/threonine residues. Gel-forming mucins are secreted by goblet cells within the gastrointestinal tract, and represent the interface between the microbial community and host tissue^11^. The prominent terminal locations of host sialic acids have made them one of the most frequently targeted carbohydrate receptors for pathogen adherence^12^. Microbes also express sialidases (also commonly referred to as neuraminidases), that cleave terminal sialic acid residues from host sialoglycan substrates. Bacterial sialidases and their mucosal sialoglycan targets contribute to host–microbe interactions at every mammalian mucosal surface. Sialidases have been proposed to promote bacterial survival in mucosal niche environments via (i) nutritional benefits of sialic acid catabolism, (ii) unmasking of cryptic host ligands used for adherence, (iii) participation in biofilm formation and (iv) modulation of immune function^13^. Moreover, removal of sialic acid from sialomucin exposes the glycan moiety that can be more rapidly catabolized^11^. A number of sialidase-expressing microbes benefit from sialic acid hydrolysis via catabolism and utilization of sialic acid as a carbon source. In bacteria, the genes involved in sialic acid metabolism are usually found clustered together forming what is denominated as a Nan cluster. The canonical cluster *nanA/K/E*, first described for *Escherichia coli*^14^ involves genes encoding the enzymes *N*-acetylneuraminate lyase (NanA), kinase (NanK) and *N*-acteylmannosamine (ManAc) epimerase (NanE), converting sialic acid (Neu5Ac) into *N*-acetylglucosamine-6-P (GlcNAc-6-P), whereas the genes encoding NagA (GlcNAc-6-P deacetylase) and NagB (glucosamine-6-P deaminase) converting GlcNAc-6-P into fructose-6-P, which is a substrate in the glycolytic pathway, vary in their locations among the different genomes that encode the Nan cluster^15^. An alternative pathway for sialic acid metabolism has later been discovered in *Bacteroides fragilis,* relying on the action of an aldolase (NanL), a novel ManNAc-6-P epimerase (also named NanE; we refer to this as NanE2 to distinguish it from the *E. coli* NanE which we refer to as NanE1) and a hexokinase (RokA), converting Neu5Ac into GlcNAc-6-P, an intermediate in the common pathway for aminosugar utilization^16^. This novel sialic utilization pathway is defined by the *nanLET* cluster, where NanT is a sialic acid transporter^16^,^17^. The majority of the bacteria that encode either of these Nan clusters colonize mucus regions of the human body, such as the gut, lung, bladder or oral cavity, where sialic acid is highly abundant and can serve as a source of energy, carbon, and nitrogen^13^,^18^. Interestingly, some bacteria appear to have only partial packages of enzymes for scavenging host sialic acids. For example, *B. thetaiotaomicron* encodes a sialidase and can release free sialic acid, but lacks the Nan operon required to consume the liberated monosaccharide and does not appear capable of consuming hydrolysed sialic acids^19^. On the other hand, *Salmonella typhimurium* and *Clostridium difficile* encode the Nan operon but each lacks the sialidase^18^, and have been suggested to rely on other sialidase-producing organisms to acquire this potential nutrient source^15^,^20^.

We recently showed that *R. gnavus* ATCC 29149 encodes a complete Nan cluster (NanA/K/E) as well as the sialidase required for sialic acid liberation from host sialylated substrates (NanH) and a putative sialic acid ABC transporter (Supplementary Fig. 1), and that the whole cluster was induced when the cells were grown in the presence of α2-3-linked sialic acid lactose (3′SL) or mucin^21^. The sialidase and Nan cluster is absent from the genome of *R. gnavus* E1, which is unable to grow on mucin as a sole carbon source^21^. Taken together, these data indicate that sialic acid metabolism is key to the ability of *R. gnavus* strains to utilize mucin as a nutrient source, which is in agreement with earlier studies on mucin degradation in the human colon ecosystem^22^,^23^,^24^. However, the bacteria (ATCC 29149 and E1) could not grow on sialic acid as sole carbon source^21^. Here we functionally and structurally characterize *R. gnavus* ATCC 29149 sialidase (*Rg*NanH), demonstrating that the enzyme is an intramolecular *trans*-sialidase (IT-sialidase) producing 2,7-anhydro-Neu5Ac selectively from α2-3-linked sialic acid substrates, the first one reported in a gut commensal microbe suggesting an unprecedented mechanism underpinning adaptation of gut bacteria to the sialylated-rich mucosal environment.

## Results

### *Rg*NanH produces 2,7-anhydro-Neu5Ac from α2-3-linked Neu5Ac

**Figure 1:**
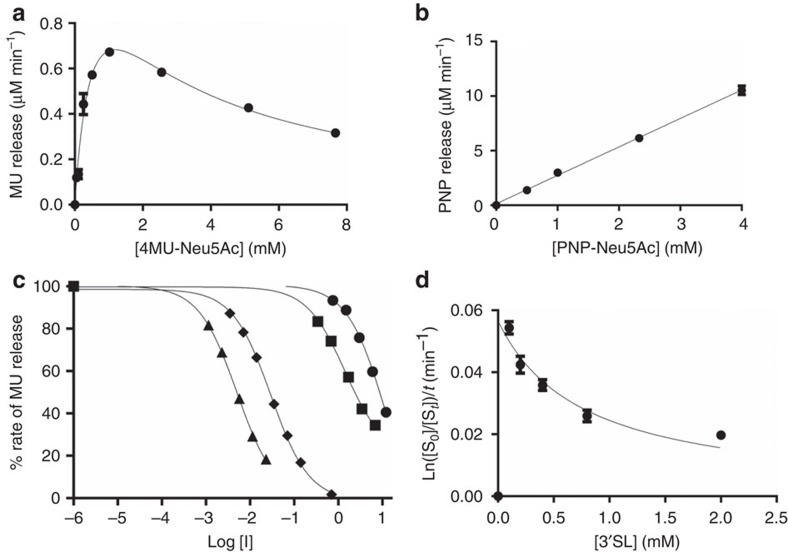
Kinetic analysis of the hydrolysis of sialylated substrates by *Rg*NanH in presence or absence of inhibitors. Substrate (**a**) 4MU-Neu5Ac or (**b**) PNP-Neu5Ac was incubated with *Rg*NanH (0.22 mM and 0.43 nM, respectively) at 37 °C, pH 6.5 and the release of product measured using a plate reader. (**c**) Inhibition of *Rg*NanH (1.00 nM) incubated with 4MU-Neu5Ac (0.51 mM) by zanamivir (●), Neu5Ac2en (▪), siastatin B (▴) and OC (♦), the rates are normalized to a % of the uninhibited rate. [I] is the inhibitor concentration. (**d**) 3′SL was incubated with *Rg*NanH (2.25 nM) and the release of product measured by HPAEC-PAD. Experiments were done at least in duplicate (usually triplicate) and the error bars show the s.e.m.

To determine the substrate specificity of *R. gnavus* ATCC 29149 sialidase (*Rg*NanH), the full-length gene was heterologously expressed in *E. coli* and the recombinant enzyme was purified to homogeneity. The enzymatic activity of the recombinant protein was first assessed using the synthetic fluorescent substrate, 2′-(4-methylumbelliferyl)-α-D-*N*-acetylneuraminic acid (4MU-Neu5Ac; Table 1). *Rg*NanH displayed a pH optimum of 6.5 and a broad temperature optimum ranging from 25 to 40 °C (Supplementary Fig. 2), consistent with the environment of the human gut. These conditions (pH 6.5, 37 °C) were used to determine the kinetic parameters of *Rg*NanH against the synthetic substrates 4MU-Neu5Ac and 2-O-(p-nitrophenyl)-α-D-*N*-acetylneuraminic acid (PNP-Neu5Ac; Fig. 1a,b and Table 1). *Rg*NanH showed 6.21 × 10^3^ min^−1^ *k*_cat_ and 0.59 mM *K*_M_ against 4MU-Neu5Ac, but a high *K*_M_ and low activity against PNP-Neu5Ac.

The activity of *Rg*NanH against 4MU-Neu5Ac was further tested in the presence of a range of known neuraminidase inhibitors (Supplementary Fig. 3). *Rg*NanH showed moderate inhibition by 2-deoxy-2, 3-didehydro-D-*N*-acetylneuraminic acid (Neu5Ac2en; IC_50_ of 1.4 mM) and a lower inhibition by its derivative 4-guanidino-Neu5Ac2en (zanamivir; IC_50_ of 11.89 mM). Oseltamivir carboxylate (OC) was substantially more effective with an IC_50_ of 30 μM (Fig. 1c and Table 2). Siastatin B was the best inhibitor of *Rg*NanH with an IC_50_ of 5 μM (Fig. 1c and Table 2). *Rg*NanH was not inhibited by lactose (Lac) or Neu5Ac (Supplementary Fig. 4).

**Figure 2:**
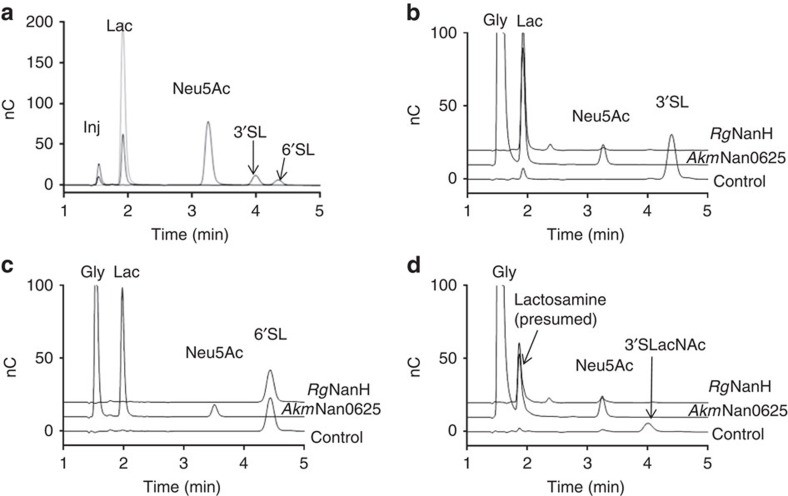
Specificity of *Rg*NanH towards sialylated oligosaccharides. The enzymes were incubated with sialylated substrates at 37 °C, pH 6.5, overnight and the reaction products were analysed by HPAEC-PAD. Control reactions without enzyme were also performed. The injection peak is marked (inj). Glycerol (gly) was also present in *Akm*Nan0625. (**a**) Standards (Lac, Neu5Ac, 3′SL and 6′SL), (**b**) 3′SL, (**c**) 6′SL and (**d**) 3'-α-sialyl-*N*-acetyllactosamine (3′SLacNAc). *Akm*Nan1835 has a similar profile to *Akm*Nan0635.

Preference of *Rg*NanH in cleaving either α2-3- or α2-6-linked sialic acid was assessed by incubation of the enzyme with 3′SL and 6′-sialyllactose (6′SL), which contain α2-3- and α2-6-linked sialic acid, respectively (Fig. 1d and Table 3). The enzyme showed a *K*_M_ for 3′SL below detection limit, indicative of a very high affinity of the enzyme for this substrate and an estimated *k*_cat_ of 25.7 min^−1^, whereas no enzymatic activity was observed in presence of 6′SL, demonstrating exclusive substrate specificity of the enzyme for α2-3 linkages. The higher activity (*k*_cat_) of the enzyme for 4MU-Neu5Ac compared with 3′SL may be due to MU acting as a better leaving group. The enzyme showed substrate inhibition with both synthetic and natural substrates (Fig. 1a,d and Table 1), with a lower *K*_i_ for the 3′SL (0.76 mM), compared with 4MU-Neu5Ac (2.37 mM). In contrast, two sialidases *Akm*Nan0625 and *Akm*Nan1835 from *Akkermansia muciniphila,* used as controls, were active against both 3′SL and 6′SL (Fig. 1a,b). The substrate and product specificity of the enzymes were further monitored by HPAEC-PAD (high-performance anion exchange chromatography-pulsed amperometric detection; Fig. 2 and Table 3) using substrates ranging from the monosaccharide galactose to the branched trisaccharide Lewis X conjugated to sialic acid with either α2-3 or α2-6 linkages. While the *A. muciniphila* sialidases released Neu5Ac from all substrates tested, *Rg*NanH activity was only observed for α2-3-linked sialyl-oligosaccharides, as observed by the disappearance of the substrate peak and the appearance of a lower-molecular weight peak that may correspond to the desialylated substrate. However, no free Neu5Ac was observed from the action of *Rg*NanH.

**Figure 3:**
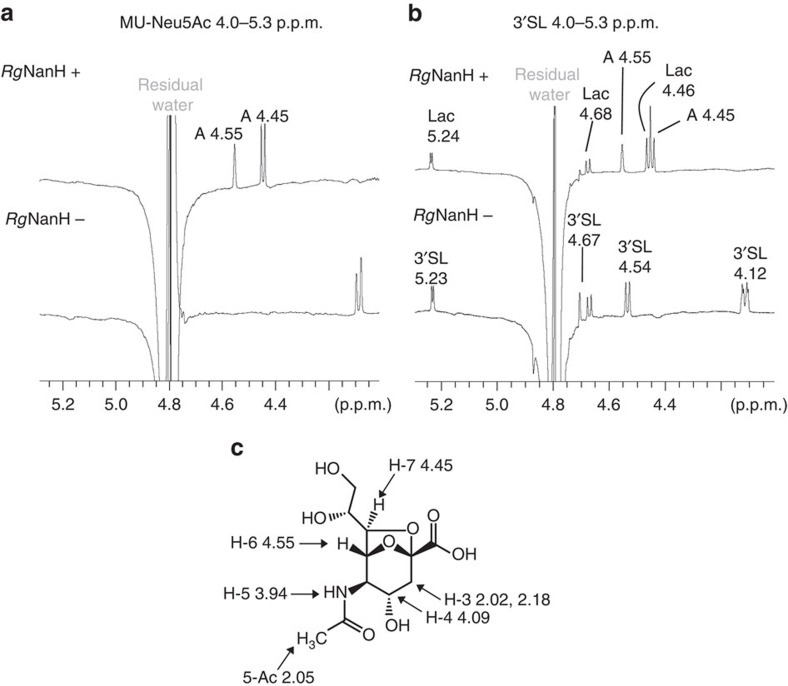
H1 NMR analysis of *Rg*NanH reaction products in presence of 4MU-Neu5Ac or 3′SL. *Rg*NanH was incubated with (**a**) 4MU-Neu5Ac or (**b**) 3′SL at 37 °C, pH 6.5, overnight and the reaction mixture analysed by H1 NMR. Control reactions without enzyme (‘—', lower trace) were carried out in parallel with the reaction containing enzyme (‘+', upper trace). ‘A' corresponds to 2,7-anhydro-Neu5Ac, see **c** for chemical structure and NMR shifts.

The products of the reaction were thus further monitored by ^1^H nuclear magnetic resonance (NMR). The spectra clearly showed the presence of two ^1^H NMR signals at 4.56 and 4.45 p.p.m. (Fig. 3). These peaks are characteristic of 2,7-anhydro-α-*N*-acetylneuraminic acid (2,7-anhydro-Neu5Ac) and arise from the protons in positions 6 and 7 (Fig. 3a). Peaks corresponding to 2,7-anhydro-Neu5Ac were observed when *Rg*NanH was incubated in presence of 4MU-Neu5Ac (Fig. 3a) or 3′SL (Fig. 3b). No changes in the peaks corresponding to 2,7-anhydro-Neu5Ac were observed after incubation of *Rg*NanH with sialylated substrates for up to 24 h at 37 °C, and no signal corresponding to free sialic acid could be detected, as also observed with the HPAEC-PAD experiments, indicating no spontaneous conversion of 2,7-anhydro-Neu5Ac to Neu5Ac. Conversely, there was no evidence of spontaneous conversion of Neu5Ac to 2,7-anhydro-Neu5Ac by NMR under the experimental conditions tested. The signals of 2,7-anhydro-Neu5Ac and their chemical shifts are shown in Supplementary Table 1. This product was absent in control experiments using *A. muciniphila* sialidases (*Akm*Nan0625 and *Akm*Nan1835) or in absence of enzyme, confirming the specificity of the enzymatic reaction (Supplementary Fig. 5). These data indicate that *Rg*NanH produces 2,7-anhydro-Neu5Ac rather than Neu5Ac from sialylated oligosaccharides.

The ability of *Rg*NanH to hydrolyse sialic acid from glycoproteins was investigated by incubation of the enzyme with human α-1-acid glycoprotein (AGP) and fetuin (Fet) both of which are known to contain α2-3 sialyl linkages, while asialo-Fet was also included as a control. *Rg*NanH showed activity against AGP and Fet, as monitored by ^1^H NMR with the detection of peaks corresponding to 2,7-anhydro-Neu5Ac but not Neu5Ac, whereas no change was observed in the control reaction with asialo-Fet in presence or absence of *Rg*NanH (Supplementary Fig. 6).

Taken together, these data demonstrate that *Rg*NanH encodes an IT-sialidase, producing 2,7-anhydro-Neu5Ac selectively from α2-3-linked sialic acid substrates.

### *Rg*NanH shares structural homology with IT-sialidases

*Rg*NanH sequence encodes a three domain modular protein with an N-terminal lectin-like domain (L-domain) classified as a member of the carbohydrate-binding module family 40 (CBM40; www.cazy.org), a catalytic domain (N-domain) classified as a member of the glycoside hydrolase family 33 (GH33; www.cazy.org) and a domain inserted into the catalytic domain (I-domain). To date, only two enzymes with IT-sialidase activity have been reported, NanL from *Macrobdella decora* (North American leech)^25^ and NanB from the human pathogen *Streptococcus pneumoniae*^26^. *Rg*NanH shares ∼75% and ∼42% sequence identity over the whole protein length with NanL and NanB, respectively, and the same multidomain architecture. Homology is greatest in the catalytic domain (excluding the I-domain), showing ∼81% and ∼46% identity with NanL and NanB, respectively. The CBM40 L-domain shares ∼67% and ∼30% identity, and the I-domain shares 64% and 43%, both respective to NanL and NanB.

**Figure 4:**
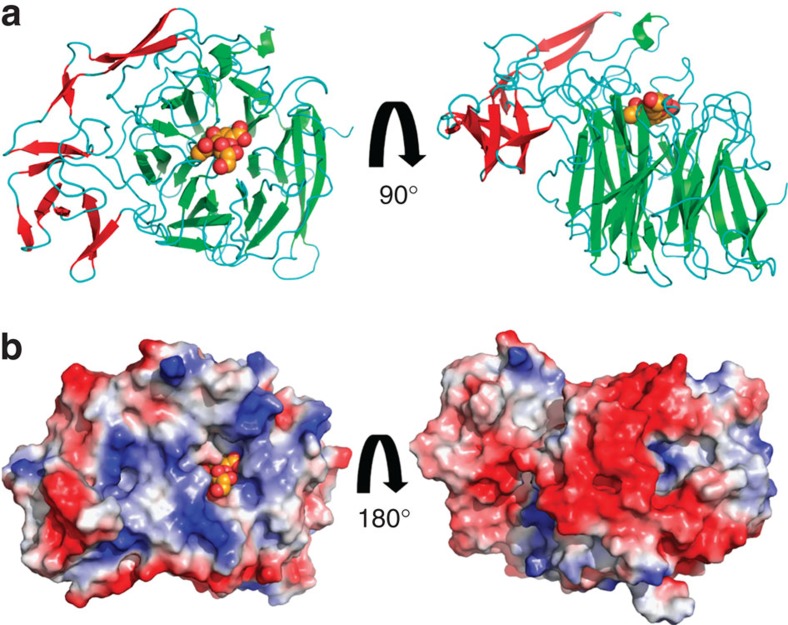
***Rg*****NanH catalytic domain bound to Neu5Ac2en.** (**a**) Cartoon representation of the *Rg*NanH NI-domain, the catalytic N-domain is shown with green secondary structure features and the inserted I-domain is shown with red secondary structure features. Neu5Ac2en is shown bound in the active site. (**b**) The *Rg*NanH NI-domain with an electrostatic surface applied. Neu5Ac2en is shown bound in the active site.

**Figure 5:**
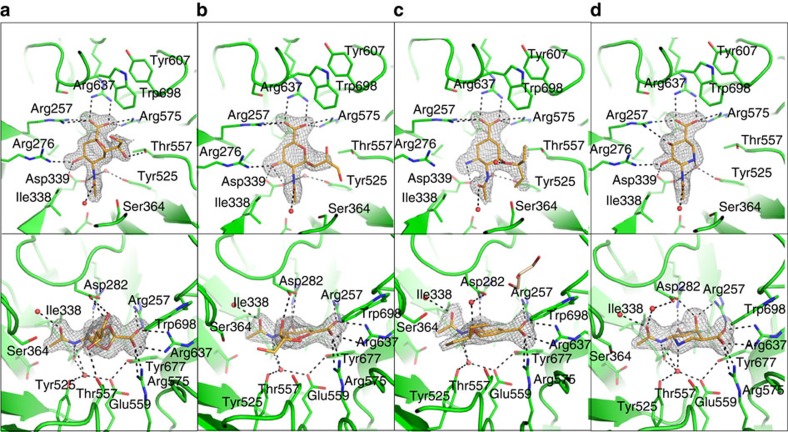
The *Rg*NanH active site bound in complex with 2,7-anhydroNeu5Ac and sialidase inhibitors. Cartoon representation of the *Rg*NanH active site complexed with (**a**) 2,7-anhydro-Neu5Ac, (**b**) Neu5Ac2en, (**c**) OC and (**d**) siastatin B. Bound ligands are shown in orange with the unbiased Fo-Fc map contoured to *σ*=3. Relevant residues are highlighted as sticks and water molecules as red spheres. Black dashed lines represent the hydrogen-bonding network. The lower panel is related to the upper panel by a rotation along the *z* axis of −90° followed by a rotation along the *x* axis of −45°.

To investigate the structural basis for the IT-sialidase reaction by *Rg*NanH, we determined the crystal structure of *Rg*NanH catalytic domain, free and in complex with the ligands identified above. *Rg*NanH catalytic domain (residues 243–723) was heterologously expressed and purified, and the crystal structure of the N-domain with the inserted I-domain (NI-domain) was solved to a maximum resolution of 1.71 Å (Table 4). As shown in Fig. 4a, the N-domain adopts the canonical sialidase six-bladed β-propeller fold, which is shared with NanL^27^ (PDB 2SLI) and NanB^28^ (PDB 2VW0). The N-domain contains four asparagine boxes, one in each of the first four β-propeller blades. These are common structural motifs in bacterial sialidases and form β-hairpin loops between β-strands three and four of a β-propeller blade, providing blade-to-blade interactions^29^. There is an extended β-hairpin loop in the equivalent position in blade five. The I-domain is formed by a loop extended from between two blades of the propeller and is primarily comprised of β-strands (Fig. 4a). The enzyme surface displays a significant charge bias, with the active site face being generally positive and the opposite face substantially negative (Fig. 4b). Soaking of the NI-domain crystals with 3′SL resulted in substrate turnover, leading to a protein ligand complex with 2,7-anhydro-Neu5Ac bound into the active site (Fig. 5). *Rg*NanH active site presents the classical features of bacterial sialidases, shared by hydrolytic and *trans*-sialidases (Fig. 5a). These include an arginine triad formed by Arg257, Arg637 and Arg575; a catalytic pair formed by Glu559 and Tyr677; a general acid base, Asp282; and a pocket to accommodate the *N*-acetyl group. As shown in Fig. 5a, the arginine triad forms electrostatic interactions with the 2,7-anhydro-Neu5Ac carboxylate group, orientating the ligand in the active site. Asp282 forms hydrogen bonds with the newly formed ethylene glycol group. The ligand *N*-acetyl group sits in a pocket between Ser364 and Ile338 with the amine providing hydrogen-bonding interactions to Asp339, and Glu559 and Tyr525 via a buried water molecule. The carbonyl group of the *N*-acetyl functional group may also interact with Asp282 via a water molecule.

The formation of the intramolecular sialosyl linkage specific to IT-sialidases has been proposed to be owing to the positioning of a threonine underneath the glycerol group of the sialic acid substrate, forcing it into an axial position from which it can attack the ring carbon C2 (ref. 30). In *Rg*NanH, this mechanism would appear to be conserved through Thr557. Furthermore, there is a hydrophobic stack formed by Tyr607 and Trp698 in front of the active site (Fig. 5a). This feature is likely to be responsible for the specificity of IT-sialidases for α2-3-linked substrates and also for creating a generally hydrophobic region of the active site, favouring nucleophilic attack by the glycerol group rather than an incoming water molecule^30^.

To gain further insights into the inhibition profile of this enzyme, crystal structures of *Rg*NanH in complex with inhibitors tested enzymatically were investigated. Complexes with Neu5Ac2en, OC and siastatin B, bound in the active site, were solved to 2.00, 2.01 and 1.94 Å, respectively (Table 4, Fig. 5b–d and Supplementary Fig. 7). It was not possible to obtain a crystal structure of zanamivir in complex with the enzyme, probably owing to its low affinity for *Rg*NanH (IC_50_ of ∼12 mM). Neu5Ac2en (IC_50_ of 1.4 mM) bound to *Rg*NanH active site in a half-boat conformation, planar around the C2 (Fig. 5b). Most protein–inhibitor interactions were similar to those observed with 2,7-anhydro-Neu5Ac, including the carboxylic acid group to the arginine triad, hydroxyl O4 to Arg276 and Asp339, and those made by the *N*-acetyl group. There is a hydrogen bond between the first hydroxyl of the glycerol group (O7) and Asp282 (Fig. 5b), which is similar to the interaction observed between Asp282 and the ethylene glycol group of 2,7-anhydro-Neu5Ac. OC (IC_50_ of ∼30 μM) is a substantially more potent inhibitor of *Rg*NanH than Neu5Ac2en. Based on a cyclohexene scaffold rather than didehydropyran, OC has an amine at position C4 in comparison with the hydroxyl of Neu5Ac2en and a pentyl ether group in place of the glycerol group (Supplementary Fig. 3). The C4 amine formed hydrogen bonds with *Rg*NanH Asp282 and Asp339 (Fig. 5c). OC is shifted out of the active site by ∼0.6 Å, as compared with Neu5Ac2en, which may be due to repulsive effects between the inhibitor amino group and Arg276. The ether oxygen of the pentyl ether functional group may interact with Asp282 via a water molecule.

Siastatin B (IC_50_ of ∼5 μM) was the most potent inhibitor tested. Siastatin B is based on an iminosugar scaffold with carboxylic acid, hydroxyl and *N*-acetyl functional groups at equivalent positions to Neu5Ac, an additional hydroxyl group is present at position 3 (Supplementary Fig. 3). The siastatin B inhibitor bound to the active site in a chair conformation (Fig. 5d). Like Neu5Ac2en, the carboxylic acid group of siastatin B interacts with the arginine triad, and the O4 hydroxyl with Arg276 and Asp339. Furthermore, the axial C3 hydroxyl of siastatin B formed hydrogen bonds to Asp282 and Arg257 of the arginine triad. Asp282 may interact with the carbonyl of the ligand *N*-acetyl group via a water molecule. This was also observed in the 2,7-anhydro-Neu5Ac complex. Both the nitrogen of the carbohydrate ring and of the *N*-acetyl group formed hydrogen bonds to a buried water molecule, resulting in a more extensive hydrogen-bonding network in this region than seen in the Neu5Ac2en and OC complexes.

### IT-sialidases are overrepresented in IBD gut metagenomes

To assess the prevalence of IT-sialidase-positive bacteria across the human gut microbiota, initial assemblies of sequenced metagenomes of human stool samples from 99 healthy subjects and 25 IBD patients^10^ were examined for the presence of IT-sialidases. We used profile hidden Markov models (pHMMs) to search the protein sequences resulting from translating the assembled coding sequences (14.1 million in all, with a mean length of 637 bp). All subjects' metagenomes were positive for a sialidase gene (mean 30 coding sequences per subject). The IT-sialidase was more scarcely detected, being present in 10.5% of all subjects, with a mean of 1.5 sequences per positive subject. However, of the non-IBD subjects, 8.1% were positive for IT-sialidase in contrast to 20% of the smaller group of IBD subjects. In the two groups, respectively, 1 in 830,000 and 1 in 422,000 of all the coding sequences were identified as IT-sialidase (Table 5). These data are consistent with an overrepresentation of IT-sialidases in IBD patients.

To further investigate the nature of the bacteria species possessing the IT-sialidase-encoding gene (*Rg*NanH), in addition to the known Nan clusters, we performed a bioinformatics analysis using the same pHMMs (see Methods section for details). We found that the genomes of 488 bacterial strains (6% of the 8,126 analysed), representing 44 species, had IT-sialidase hits, of which 94% (457 strains), representing 32 species, tested positive for potential NanA-, NanK- and NanE1-encoding genes within a cluster spanning up to 15 contiguous gene loci. These strains represent 18% of all genomes with this cluster. A further six IT-sialidase-positive species (all with genomes in draft form) matched these three genes but not in a cluster. Four of the IT-sialidase species were positive for homologues of the NanL, NanE2, RokA components of the *B. fragilis* pathway (in total, 1,796 strains possessed these components). All strains positive for the *B. fragilis* components lacked a match to NanE1 (Supplementary Table 2). A total of 3,427 strains were found positive for either the NanL/RokA/NanE2 set of genes or the NanA/K/E1 cluster or both (if the latter genes are permitted to be present but not necessarily in a cluster then this rises to 4,127). Of these, 460 (13%) have an IT-sialidase gene. The CBM40 domain was associated with 75% of the IT-sialidases. Interestingly the IT-sialidase-positive strains from species known to occur in the human gut seemed restricted to Firmicutes, in particular, Clostridiales and Lactobacillales (Supplementary Table 2).

## Discussion

Sialidases are a large group of enzymes that catalyse the cleavage of terminal sialic acids from complex carbohydrates on glycoproteins or glycolipids. Based on their substrate specificity and catalytic mechanism, sialidases can be separated into three different classes. Hydrolytic sialidases cleave the glycosidic bond of terminal sialic acids and release free sialic acid, whereas *trans*-sialidases transfer the cleaved sialic acid to other glycoconjugates; according to the Enzyme Commission, both classes belong to exo-α-sialidases (EC 3.2.1.18). Hydrolytic sialidases usually have wide substrate specificity and cleave α2-3-, α2-6- and α2-8-linked terminal sialic acids, whereas *trans*-sialidases have a preference for α2-3-linked substrates. The third class is the IT-sialidase (EC 4.2.2.15) that is strictly specific for α2-3-linked sialic acids and produces 2,7-anhydro-Neu5Ac (ref. 31). To date, only NanL from *M. decora* and NanB from *S. pneumoniae* have been assigned to this third class^25^,^26^. These enzymes are unique in that they catalyse an IT-reaction in which the O7-hydroxyl group of the bound sialic acid glycerol group attacks the positively charged C2 atom of the oxocarbenium intermediate. This altered reaction pathway leads to the release of 2,7-anhydro-Neu5Ac instead of Neu5Ac, the reaction product for hydrolytic sialidases. Here we have shown that *Rg*NanH is a novel member of this class, the first one identified and characterized in members of the human gut microbiota. The enzyme showed strict specificity towards α2-3 glycosidic substrate linkages as tested by HPAEC and ^1^H NMR using a range of natural substrates from disaccharides to branched tetrasaccharides. The production of 2,7-anhydro-Neu5Ac in the reaction with 3′SL and synthetic substrates 4MU-Neu5Ac and PNP-Neu5Ac confirmed that *Rg*NanH is an IT-sialidase. Furthermore, human α-1-AGP and Fet were both substrates of *Rg*NanH, in agreement with the presence of α2-3 sialic acid linkages in these proteins; Neu5Ac-α2-6-Gal, Neu5Ac-α2-3-Gal as well as Lewis X epitopes are found in AGP^32^, whereas Fet also contains sialylated α2-6-Gal and α2-3-Gal epitopes^33^. The *Rg*NanH crystal structures showed active site features associated with the IT-sialidase class. Of particular importance is the conservation of Thr557, which seems to sterically force the substrate glycerol group into a position from where it can attack the C2 atom, and of the Tyr607 and Trp698 hydrophobic stack, proposed to be responsible for the observed substrate specificity and also the maintenance of a desolvated active site promoting nucleophilic attack by the glycerol group^27^.

*Rg*NanH displayed activity against all α2-3-linked substrates tested. So far, the active site has been considered to be the pocket accommodating the terminal sialic acid residue of the substrate. However, the reaction rate is greatly influenced by the leaving aglycone, as shown by the variation in *K*_M_ and efficiency of cleavage (*k*_cat_/*K*_M_) between 4MU-Neu5Ac and PNP-Neu5Ac, even though both the fluorophore and chromophore have similar leaving abilities (p*K*_a_ 7.79 and 7.23, respectively). 3′SL also displays much tighter apparent affinity than the synthetic substrates. Similar results have also been observed in other sialidases. For example, NanC, an α2-3-linkage-specific sialidase from *S. pneumoniae* that shares 42% identity with *Rg*NanH, shows a preference to LacNAc- compared with Lac-based substrates, and demonstrates reduced activity towards fucosylated glycans^34^. In the *Trypanasoma cruzi trans*-sialidase, 4MU-Neu5Ac is a much poorer substrate than 3′SL, and molecular dynamics simulations suggest that MU, the 4MU-Neu5Ac leaving group, is unable to form the same favourable interactions as Lac^35^. It is probable that additional interactions between *Rg*NanH and the aglycone may tune the enzyme specificity to complex substrates. Furthermore, both 3′SL and 4MU-Neu5Ac showed substrate inhibition, suggesting a potential mechanism for regulation of this enzyme activity. To the best of our knowledge, substrate inhibition has not been reported for other sialidases, but there is a paucity of kinetic data, particularly for IT-sialidases.

Siastatin B was the most effective inhibitor of *Rg*NanH with an IC_50_ of ∼5 μM, compared with zanamivir (IC_50_ of ∼12 mM), Neu5Ac2en (IC_50_ of 1.4 mM) and OC (IC_50_ of 30 μM). Our crystal structures of *Rg*NanH inhibitor complexes, including the first published structure of a complex between siastatin B and a sialidase, provide a structural explanation for the differences in inhibition between the chemical inhibitors tested. Neu5Ac2en, an analogue of Neu5Ac dehydrated at C2, is a general sialidase inhibitor that inhibits both viral and bacterial sialidases^36^,^37^, including the hydrolytic sialidase expressed by *B. thetaiotaomicron*^38^. It has been proposed that Neu5Ac2en is a poor inhibitor and transition state mimic of IT-sialidases because the glycerol group is constrained by the C2=C3 bond and is unable to occupy the axial position required for catalysis^27^, as also seen in the *Rg*NanH–Neu5Ac2en complex (Fig. 5b). OC and zanamivir are variants of Neu5Ac2en originally designed to improve inhibition of the viral neuraminidase from influenza. In the case of OC, these modifications led to an improvement in inhibition of *Rg*NanH by ∼50-fold (IC_50_: 1.4 mM to 30 μM). This is likely owing to enhanced interactions from the C4 amine group, as seen in the complex structure (Fig. 5c). In contrast, Neu5Ac2en makes a direct hydrogen bond to Asp282 via the hydroxyl O7. This interaction is not present in the complex with OC, although there may be a water-mediated interaction from Asp282 to the ether oxygen. In viral neuraminidases, and hydrolytic sialidases such as NanA from *S. pneumoniae*, there are substantial hydrophobic interactions with the pentyl ether functional group of OC^36^,^37^; these interactions are not present in *Rg*NanH, which may explain why OC is less potent towards IT-sialidase. Zanamivir was a very poor inhibitor of *Rg*NanH, which is not surprising since the inhibitor has a much bulkier guanidino substitution at the C4 position, which, based on the above complex crystal structures, would clash with Arg276 and Asp339. The complex between siastatin B and *Rg*NanH suggests a structural explanation for its effective inhibition of the enzyme (Fig. 5d). Siastatin B adopts a chair conformation rather than the more planar, half-boat Neu5Ac2en conformation. Furthermore, in comparison with Neu5Ac2en and OC, siastatin B makes a more extensive hydrogen-bonding network underneath the ligand via the buried water. Siastatin B also makes extensive interactions through the O3 hydroxyl to Asp282 and Arg257. Finally, although both Neu5Ac2en and siastatin B form hydrogen bonds to Asp282 (Neu5Ac2en via the glycerol group), siastatin B achieves such interaction without paying the entropic penalty required to avoid a steric clash between the glycerol group and Thr557. Overall, the inhibitor data showed considerable differences between *Rg*NanH and viral neuraminidases, whether this is due to the nature of the enzyme, IT-sialidase versus hydrolytic sialidase, or due to its bacterial versus viral origin remain to be demonstrated.

Our biochemical assays highlighted differences in sialylated substrate specificity between *R. gnavus* and other gut mucin-degrading bacteria, such as *A. muciniphila*, which is a dedicated mucus utilizer that has found to increase intestinal permeability in mice^39^,^40^. Many enteric commensal and pathogenic bacteria can utilize sialic acids from their hosts owing to the presence of a Nan cluster^18^,^41^, but not all have the ability to release sialic acid from glycoproteins. Among gastrointestinal commensals, Bacteroidetes species are found at high abundance and many of them express sialidases in culture^42^. However, some bacteria, such as *B. thetaiotaomicron* encode the sialidase required to cleave and release this terminal sugar from the mucosal glycoconjugates, but do not encode a sialic acid lyase/aldolase homologue and lack the catabolic pathway (that is, the Nan clusters) required to consume the liberated monosaccharide. Presumably, the release of sialic acids allows *B. thetaiotaomicron* to access highly coveted underlying carbohydrates in the mucus^43^,^44^. *B. fragilis* or *E. coli* on the other hand possesses the complete pathway of sialic acid catabolism including the hydrolytic sialidase gene^16^,^45^. Both *B. fragilis* and *E. coli* catabolic genes are upregulated in response to available free sialic acid^45^. A recent study reported that mice monoassociated with *B. thetaiotaomicron* exhibited a significantly higher concentration of free Neu5Ac versus germ-free mice, consistent with the ability of *B. thetaiotaomicron* to liberate, but not consume, the monosaccharide, whereas colonization of mice with *B. fragilis*, which is able to catabolize Neu5Ac, did not result in increased free sialic acid^20^. This repartition of free sialic acid in the gut is important as some bacterial pathogens such as *S. typhimurium* and *C. difficile* do not encode sialidases and thus rely on the sialic acid liberated by the resident microbiota to expand in the mucosal environment^46^,^47^. This cross-feeding activity has also been reported between members of Bifidobacteria, for example, *Bifidobacterium breve* UCC2003 (containing a functional Nan cluster for sialic utilization) can utilize sialic acid released by the sialidase activity of *B. bifidum* PRL2010 (ref. 48). *R. gnavus* ATCC 29149 is different to the above as it possesses the complete Nan cluster and an IT-sialidase-producing 2,7-anhydro-Neu5Ac instead of Neu5Ac from α2-3-linked sialic acid substrates, which is consistent with the bacteria ability to grow on 3′SL but not on sialic acid, Lac or 6′SL and the induced expression of the Nan cluster and *Rg*NanH on 3′SL (ref. 21). Our bioinformatics analysis revealed that the presence of IT-sialidases is shared by other members of the gut microbiota, in particular *Blautia hansenii* and *R. torques*, all 10 strains of *C. perfringens* with available genome data, *C. sp.* 7 2 43 FAA, *C. celatum*, *C. nexile* and *C. spiroforme*, three unclassified Lachnospiraceae, >100 strains of *S. agalactiae* and three of the genome-sequenced publicly available *Lactobacillus salivarius* strains. Of 1,165 strains testing positive for a sialidase and the Nan clusters, 40% (457 strains) tested positive specifically for the IT-sialidase (the remainder matched the sialidase domain but lacked the I-domain). The detection of IT-sialidase homologues in at least 11% of gut metagenomes of a population of diseased and healthy humans is in line with this analysis, showing that this enzyme is widespread across gut bacteria, especially in Firmicutes. The specific niche colonization of these bacteria may reflect an adaptation to particular mucus glycosylation profiles. The *R. gnavus* mucin-utilization enzymatic profile, which is mainly based on the release and use of sialic acid to support growth^21^, appears particularly well adapted to digest mucin with short chains terminated by sialic acid, such as sialyl-Tn-antigen, which is found in higher proportion in IBD patients^49^. On the contrary, other mucin degraders such as *A. muciniphila* or *B. thetaiotaomicron* cannot utilize sialic acid as carbon source, so will be disadvantaged by the mucin glycosylation profile of IBD patients, while more adapted to utilize complex mucin glycan structures^50^ that require the synergistic action of several glycoside hydrolases^51^. Furthermore, the IT-sialidase may provide gut microbes such as *R. gnavus* ATCC 29149 with an additional competitive nutritional advantage, allowing the bacteria to thrive within mucosal environments by scavenging sialic acid from host mucus in a form, 2,7-anhydro-Neu5Ac, which may not be readily accessible to other members of the gut microbiota, thus limiting loss to other microbiota residents and/or exploitation by enteric pathogens. This ‘selfish' model of mucosal glycan utilization could contribute to the disproportionate representation of *R. gnavus* reported in ulcerative colitis and Crohn's disease patients^9^,^52^,^53^. It is interesting to note that *R. torques*, predicted to encode an IT-sialidase, is also a mucin degrader frequently associated with conditions such as intestinal disorders, including IBD and irritable bowel syndrome, and other health problems such as autism spectrum disorders^9^,^53^,^54^,^55^. This contrasts with the sialidases *Akm*Nan0625 and *Akm*Nan1835 from the commensal mucin degrader *A. muciniphila*, the amount of which has shown to be inversely correlated with that of *R. torques* in ulcerative colitis and Crohn's disease mucosa^9^,^47^. Furthermore, the overrepresentation of IT-sialidase-encoding genes in the metagenome of IBD patients suggests that IT-sialidase-encoding bacteria may be particularly adapted to this particular mucosal niche. Future studies in these systems should define how IT-sialidase action on specific sialoglycans influences pathological, commensal and/or symbiotic host–microbe relationships within the complex sialoglycan-rich environment of the human gut.

## Methods

### Reagents

All chemicals were obtained from Sigma (St Louis, USA) unless otherwise stated. The *A. muciniphila* sialidases, termed here *Akm*Nan0625 and *Akm*Nan1835, were obtained by His-Tag overexpression of their genes (Amuc_0625 and Amuc_1835, respectively)^56^ in *E. coli* BL21(DE3) and subsequent purification to homogeneity by immobilized metal affinity chromatography as described below. The sialylated oligosaccharides and OC were obtained from Carbosynth (Berks, UK).

### Cloning, expression and purification of full-length sialidases

The full-length *Rg*NanH sialidase excluding the signal sequence (residues 1–25) was cloned into the pOPINF expression system^57^, introducing an His-tag at the N terminus. The primers used were as follows: forward 5′-AAGTTCTGTTTCAGGGCCCGCAAGAGGCCCAGACAGAT-3′, reverse 5′-ATGGTCTAGAAAGCTTTATGGTTGAACTTTCAGTTCATC-3′. DNA manipulation was carried out in *E. coli* XL1 Blue (New England BioLabs, Boston, USA). Sequences were verified by DNA sequencing by Eurofins (Ebersberg, Germany). *E. coli* BL21 (New England BioLabs) cells were transformed with the recombinant plasmid harbouring the sialidase gene according to manufacturer's instructions. For small-scale expression, the recombinant cells were grown to an OD_600_ of 0.6 in Luria-Bertani Broth (10–50 ml) and then induced with 1 mM isopropyl β-D-1-thiogalactopyranoside overnight at 22 °C. The cells were harvested by centrifugation at 10,000*g* for 20 min. Large-scale expression (1 l) was carried out in ‘Terrific Broth Base with Trace Elements' autoinduction media (ForMedium, Dundee, UK) growing cells for 3 h at 37 °C and then induced at 16 °C for 48 h. The cells were harvested by centrifugation at 10,000*g* for 20 min. The His-tagged proteins were purified by immobilized metal affinity chromatography. His-bind resin was used according to the manufacturer's instructions (Novagen, Darmstead, Germany). Fractions containing the sialidase or domains were dialysed against 2 × 4 l 10 mM HEPES (pH 8.0) and concentrated using a 10-kDa MWCO Vivaspin column (Vivaspin, Goettingen, Germany) up to 6 mg ml^−1^. The protein was further purified by gel filtration using the Superdex 200 column on an Akta system (GE Health Care Life Sciences, Little Chalfont, UK). Protein purification was assessed by standard SDS–polyacrylamide gel electrophoresis using the both the NuPAGE Novex 4–12% Bis-Tris (Life Technologies, Paisley, UK) and RunBlue 12% SDS–polyacrylamide gel electrophoresis gels (Expedeon, Cambridge, UK). Protein concentration was measured with a NanoDrop (Thermo Scientific, Wilmington, USA) and using the extinction coefficient calculated by Protparam (ExPASy-Artimo, 2012) from the peptide sequence.

### Activity assays and kinetics

The purified enzymes were incubated with the substrate in buffer made from 20 mM Na_2_HPO_4_ and 20 mM NaH_2_PO_4_ adjusted to pH 6.5 containing 1 mg ml^−1^ BSA at 37 °C unless otherwise stated. For 4MU-Neu5Ac, the progression of the reaction was monitored by the release of the MU using a 96-well plate reader (BMG Labtech, Ortenberg, Germany) using fluorescence with an excitation at 340 nm and an emission at 420 nm. For determining the optimal temperature, the assay was carried out in 20 mM PBS at pH 7.4. For determining the optimal pH, the assay was carried out at 37 °C using buffer made from 20 mM Na_2_HPO_4_ and 20 mM NaH_2_PO_4_ in varying proportions to obtain the desired pH. PNP release from PNP-Neu5Ac was monitored in a similar way measuring absorbance at 405 nm every 60 s. For 3′SL, the reaction was monitored by HPAEC-PAD. An aliquot of the reaction was removed from the main reaction volume at the reaction stopped by boiling for 20 min, the enzyme was then removed by centrifugation at 17,000*g* for 10 min and filtration with a 0.2-μm filter (Millipore, Billerica, USA). The sugars were separated by HPAEC with an isocratic gradient of 100 mM NaOH and 100 mM NaAC at 1 ml min^−1^ on a CarboPac PA1 protected with a guard column and detected using PAD on a Dionex ICS5000 system (Thermo Scientific, Hemel Hempstead, UK). The column was cleaned with 10 ml of 500 mM NaOH and 500 mM NaAc, and the column was re-equilibrated with 100 mM NaOH and 100 mM NaAc. An internal standard of fucose was used to quantify the results.

For 4MU-Neu5Ac and 3′SL, the rate of hydrolysis without enzyme was not significant, thus raw data were used. However, for pNP-Neu5Ac, the rate of hydrolysis without enzyme was significant, thus the rate with enzyme was corrected by the subtraction of the rate without enzyme at each concentration of substrate. For 3′SL, the rate of reaction was calculated by plotting the amount of substrate remaining over the course of >50 % of the reaction using the following equation^58^:


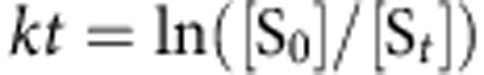


Where *k* is *k*_cat_/*K*_M_ × enzyme concentration, *t* is time, [S_0_] and [S_*t*_] are substrate concentrations at time 0 and *t*, respectively.

Kinetic data were obtained from at least duplicate (usually triplicate) experiments, and the kinetic parameters were calculated by fitting the initial raw data to the Michaelis–Menten equation using a nonlinear regression analysis programme (Prism 6, GraphPad, San Diego, USA), and error bars (s.e.m.) are shown.

For specificity tests against sialylated oligosaccharides (1 mM) and glycoproteins (1 mg ml^−1^), 1 nM of enzyme was incubated with the substrate at 37 °C, pH 6.5, overnight. For inhibition studies, the enzyme and inhibitor were pre-incubated together for 15 min at 37 °C and the reaction started by the addition of the substrate, the IC_50_ values were determined using Prism 6.

### NMR analysis

Extracts (400 μl) were mixed with 200 μl of D_2_O and 20 μl of a solution of D_2_O containing 1 mM of TSP (sodium 3-(trimethylsilyl)-propionate-d4). Samples (500 μl) were transferred into a 5-mm NMR tube for spectral acquisition. The ^1^H NMR spectra were recorded at 600 MHz on a Bruker Avance spectrometer (Bruker BioSpin GmbH, Rheinstetten, Germany) running Topspin 2.0 software, and were fitted with a cryoprobe and a 60-slot autosampler. Each ^1^H NMR spectrum was acquired with 128 scans, a spectral width of 8,012.8 Hz, an acquisition time of 2.04 s and a relaxation delay of 2.0 s. The ‘noesypr1d' presaturation sequence was used to suppress the residual water signal with a low-power selective irradiation at the water frequency during the recycle delay and a mixing time of 100 ms. Spectra were transformed with a 0.3-Hz line broadening, manually phased, baseline corrected and referenced by setting the TSP methyl signal to 0 p.p.m.

### Protein production and crystallization

The *Rg*NanH NI-domain (residues 237–723) was cloned into the pEHISTEV vector^59^ using the following primers: forward 5′-GATATCGGATCCAATATCTTTTATGCAGGAGATGC-3′, reverse 5′-TGGTGCTCGAGTTTATGGTTGAACTTTCAGTTCATC-3′. The domain was expressed and purified as described above but with the additional step of removal of the 6-histidine affinity tag by tobacco etch virus protease at a mass ratio of 1:50 overnight at 4 °C. High-purity fractions were pooled and concentrated to 36 mg ml^−1^ for storage and crystallization trials.

All crystallization experiments were carried out at 20 °C by the sitting drop, vapour diffusion method. Initial conditions were screened by the high-throughput Gryphon system (Art Robbins). Optimization was carried out manually with extensive use of serial micro-seeding. The best conditions for N-domain crystallization were as follows: 0.8 M NaH_2_PO_4_, 1.2 M K_2_HPO_4_ and 0.1 M sodium acetate (pH 4.5; condition 1); 1.2 M NaH_2_PO_4_, 0.8 M K_2_HPO_4_ and 0.1 M CAPS (pH 10.5; condition 2); and 12.5% PEG 3350 and 200 mM calcium chloride (condition 3). Typically, the crystallization drop consisted of 1 μl protein solution at 10–20 mg ml^−1^, 1 μl reservoir solution and 0.25 μl seed stock solution prepared according to Bergfors and collaborators^60^. Crystals generally appeared after 48 h and grew to full size in 72 h.

Ligand protein complexes were achieved by adding ligand stock solution in H_2_O directly to the crystallization drop. The crystallization conditions and final ligand concentrations for the relevant complexes were as follows: 15 mM Neu5Ac2en for 20 min in condition 1; 6 mM siastatin B for 20 min in condition 2; and 10 mM OC for 240 min in condition 3. To achieve the 2,7-anhydro-Neu5Ac complex, the crystals were soaked with 20 mM 3′SL for 30 min in condition 1. Crystals were cryoprotected by stroking the crystal, using a nylon loop, across the top of a 1-μl drop containing the crystallization solution with 25% glycerol.

### Structure determination and refinement

Data were collected in-house at 100 K on a Rigaku 007HFM rotating anode X-ray generator with a Saturn 944 CCD detector at a wavelength of 1.54178 Å. The data were processed with HKL2000 (ref. 61). The phase problem was solved by molecular replacement using PHASER^62^ using the structure of NanL catalytic domain (PDB 2SLI) as a search model. This was followed by manual rebuilding with COOT^63^ and refinement using REFMAC5 (ref. 64). The *Rg*NanH–siastatin B made use of the Buccaneer pipeline^65^. Ramachandran statistics in the form outliers (%)/favoured (%): 2,7-anhydro-Neu5Ac complex, 0.00/97.1; Neu5Ac2en complex 0.00/96.3; OC complex, 0.00/96.5; and siastatin B, 0.41/95.9. For a stereo view of a portion of electron density, see Supplementary Fig. 7. All molecular graphics were generated with PYMOL^66^.

### Genomic analysis of bacterial IT-sialidase

We searched for Nan clusters, sialidase-encoding genes and other associated genes in 8,236 NCBI-distributed genomes (ftp://ftp.ncbi.nlm.nih.gov) that had annotations of protein-coding sequences and their products available (May 2014), as well as two additional *R. gnavus* genomes^23^,^67^. These represent 8,126 unique strains of ∼2,800 species, comprising 28.6 million protein sequences. We used HMMER3 (http://hmmer.org) to query pHMMs of the relevant protein domain sequences; for more details see Supplementary Methods. Briefly, we refer to the seven relevant protein domains as follows: (i) NanA (*R. gnavus* NanA and homologues including *B. fragilis* NanL), (ii) NanK (*Rg*NanK and homologues), (iii) NanE1 (*Rg*NanE and homologues including *E. coli NanE*), (iv) NanE2 (*B. fragilis* NanE and homologues), (v) ‘sialidase' (the sialidase domain of *Rg*NanH and homologues but excluding the I-domain), (vi) I-domain (of *Rg*NanH and homologues where present) and (vii) CBM40 (of *Rg*NanH and homologues where present). We used pHMMs from Pfam^68^ when appropriate, but it was necessary to construct our own for (v and vi), respectively, from 984 and 132 sequence segments from the GH33 family (www.cazy.org). We also built our own model (vii) from 36 segments of these sequences, although this performed very similarly to a Pfam pHMM; note that this Pfam domain (PF02973) is named ‘sialidase' but corresponds to CBM40, and not to the actual sialidase domain (v). Note also that (v) detects conventional GH33 domains as a contiguous match as well as *Rg*NanH-type domains as a segmented match with a gap representing the location of the I-domain matched by (vi). Having located the gene loci encoding the proteins matching our pHMMs, we defined gene clusters as cases of all genes of interest (NanA/K/E) being contained within 15 consecutive loci (which may include intervening genes). In all cases, we ignored all hits with an independent *E*-value of >10^−4^.

### Metagenomic analysis of IT-sialidase prevalence

We analysed gut metagenomic data published by the MetaHIT consortium in a study of patients diagnosed with IBD and a control group^10^. We translated the assembled coding sequences (obtained from ftp://public.genomics.org.cn/BGI/gutmeta/SingleSample_GenePrediction) of the metagenomes of the 125 human subjects (99 with no IBD and 25 with IBD) described in Supplementary Table 1 of reference before searching for pHMMs (v and vi) using HMMER3 as before. Further details are in the Supplementary Information.

## Additional information

**Accession Numbers:** Coordinates in the Protein Data Bank have been deposited with accession codes 4X4A (2,7-anhydro-Neu5Ac complex), 4X47 (Neu5Ac2en complex), 4X49 (OC complex) and 4X6K (siastatin B complex).

**How to cite this article:** Tailford, L. E. *et al.* Discovery of intramolecular *trans*-sialidases in human gut microbiota suggests novel mechanisms of mucosal adaptation. *Nat. Commun.* 6:7624 doi: 10.1038/ncomms8624 (2015).

## Supplementary Material

Supplementary Information Supplementary Figures 1-7, Supplementary Tables 1-2 and Supplementary Methods

## Figures and Tables

**Table 1:** Kinetic parameters of *Rg*NanH with sialylated substrates.

**Substrate**	***V***_**max**_ **(μM min**^**−1**^**)**	***k***_**cat**_ **(min**^**−1**^**)**	***K***_**M**_ **(mM)**	***k***_**cat**_**/*****K***_**M**_ **(min**^**−1**^** mM**^**−1**^**)**	***K***_**i**_ **(mM)**
MU-Neu5Ac	1.37±0.23	6.21 × 10^3^	0.59±0.17	1.05 × 10^4^	2.37±0.65
PNP-Neu5Ac	ND	<152*	>25†	6.07 × 10^3^	ND
3′SL	5.66 × 10^−2^±0.57 × 10^−2^	25.70	<<0.1†	>>2.57 × 10^2^*	0.76±0.23

ND, not detectable.

The s.e.m. is shown.

^*^Parameter calculated using estimated value.

^†^Estimated value.

**Table 2:** Effect of neuraminidase inhibitors on *Rg*NanH activity.

**Inhibitor**	**LogIC**_**50**_	**IC**_**50**_ **(mM)**
Zanamivir	1.08±0.07	11.89
Neu5Ac2en	0.15±0.04	1.41
Siastatin B	−2.31±0.02	4.87 × 10^−3^
Oseltamivir carboxylate	−1.53±0.02	2.96 × 10^−2^

**Table 3:** Specificity of *Rg*NanH and *Akm*Nan0625 and 1,835 against 3′- and 6′-linked sialyl-oligosaccharides.

**Substrate (common name)**	**Systematic name**	Activity*	
		***Rg*****NanH**	***Akm*****Nan0625 and** ***Akm*****Nan1835**
3′-Sialyllactose	Neu5Ac-α-2-3-Gal-β-1-4-Glc	+	+
3′-Sialyl-3-fucosyllactose	Neu5Ac-α-2-3-Gal-β1-4-[Fuc-α-1-3]-Glc	+	+
3′-α-Sialyl-*N*-acetyllactosamine	Neu5Ac-α-2-3-Gal-β-1-4-GlcNAc	+	+
3′-Sialyl Lewis X	Neu5Ac-α-2-3-Gal-β-1-4-[Fuc-a-1-3]-GlcNAc	+	+
3′-Sialyl Lewis X methyl glycoside	Neu5Ac-α-2-3-Gal-β-1-4-[Fuc-a-1-3]-GlcNAc-β-OMe	+	+
3′-Sialylgalactose	Neu5Ac-α-2-3-Gal	+	+
6′-Sialyllactose	Neu5Ac-α-2-6-Gal-β1-4-Glc	−	+
6′-α-Sialyl-*N*-acetyllactosamine	Neu5Ac-α-2-6-Gal-β-1-4-GlcNAc	−	+
6′-Sialylgalactose	Neu5Ac-α-2-6-Gal	−	+

-OMe, -O-methyl group

^*^Activity was defined as disappearance of the substrate peak and appearance of a lower-molecular weight peak, which could correspond to the desialylated substrate.

**Table 4:** Data collection and refinement statistics*.

	**2,7-Anhydro-Neu5Ac**	**Neu5Ac2en**	**Oseltamivir carboxylate**	**Siastatin B**
PDB identifier	4X4A	4X47	4X49	4X6K
				
*Data collection*				
Space group	R3	R3	R3	R3
Cell dimensions				
*a, b, c* (Å)	99.26, 99.26, 130.60	101.31, 101.31, 131.94	101.10, 101.10, 131.54	99.11, 99.11, 131.71
*α*, *β*, *γ* (°)	90.00, 90.00, 120.00	90.00, 90.00, 120.00	90.00, 90.00, 120.00	90.00, 90.00, 120.00
Resolution (Å)	92.54–1.71 (1.74–1.71)	73.05–2.00 (2.03–2.00)	72.88–2.01 (2.04–2.01)	71.91–1.94 (1.97–1.94)
*R*_merge_	0.049 (0.421)	0.064 (0.490)	0.068 (0.499)	0.066 (0.458)
*I*/σ*I*	41.72 (3.09)	25.42 (3.17)	20.32 (2.93)	25.30 (3.88)
Completeness (%)	92.90 (53.00)	99.5 (99.7)	98.3(97.20)	98.30 (94.41)
Redundancy	4.2 (2.4)	2.9 (2.6)	2.7 (2.4)	3.1 (3.0)
				
*Refinement*				
Resolution (Å)	92.54–1.71 (1.76–1.71)	73.05–2.00 (2.05–2.00)	72.88–2.01 (2.06–2.01)	71.91–1.94 (2.00–1.94)
No. of reflections	51,820	32,166	30,874	32,952
*R*_work_/*R*_free_	0.143/0.188 (0.761)	0.215/0.273 (0.778)	0.184/0.245 (0.751)	0.177/0.225 (0.79)
No. of atoms	4,411	4,080	4,272	4,281
Protein	3,836	3,820	3,803	3,798
Ligand/ion	46	25	54	18
Water	529	235	415	465
*B-*factors				
Protein	29.199	40.950	30.786	26.562
Ligand/ion	37.084	39.018	34.223	20.960
Water	44.571	43.514	38.415	35.491
R.m.s.d.				
Bond lengths (Å)	0.019	0.016	0.016	0.019
Bond angles (°)	1.891	1.787	1.802	1.923

R.m.s.d., root mean squared deviation.

^*^Values in parentheses are for the highest-resolution shell. One crystal was used for each structure.

**Table 5:** Presence of IT-sialidase in the MetaHit human samples.

**Subject group**	**No. of samples**	**Mean sequences per sample**	**Mean bp per sequence**	**Protein domain**	**% Hit sequences**	**Positive samples (that is, with ≥ 1 hit sequence)**	**% Positive samples**	**Mean hit sequences per sample**
IBD=N	99	117,359.99	636.90	Sialidase	0.0259%	99	100	30.44
				I-domain	0.0001%	8	8	0.14
IBD=Y	25	101,351.28	637.90	Sialidase	0.0268%	25	100	27.12
				I-domain	0.0002%	5	20	0.24

IBD, inflammatory bowel disease; N, negative; Y, yes.

Incidence of hits in the HMMER3 search for the sialidase and I-domains in coding sequences from the MetaHIT study^10^. Each sample consists of assembled coding sequences from the gut metagenome of a single subject designated as N or Y for IBD according to that study.
