# Supplementary material for: Discovery of intramolecular trans-sialidases in human gut microbiota suggests novel mechanisms of mucosal adaptation
Source: Nat Commun. 2015 Jul 8;6:7624. doi: 10.1038/ncomms8624 (PMC4510645; doi:10.1038/ncomms8624)
Supplement: Supplementary Information — Supplementary Figures 1-7, Supplementary Tables 1-2 and Supplementary Methods [file ncomms8624-s1.pdf]

SUPPLEMENTARY INFORMATION

Supplementary Figures

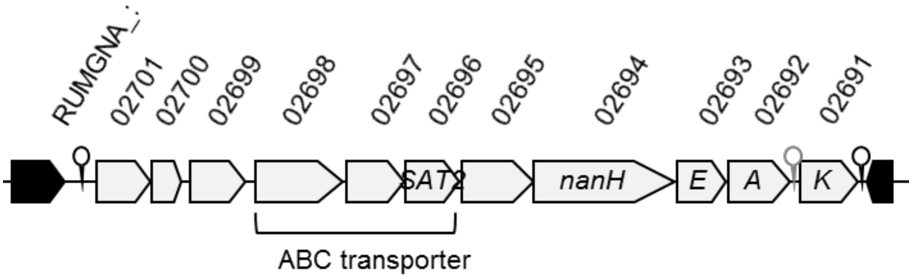

Supplementary Fig. 1 | Schematic representation of the nan locus in *R. gnavus* ATCC 29149

RUMGNA_02701 encodes a putative GDSL-like protein. RUMGNA_02700 encodes a putative sugar isomerase involved in sialic acid catabolism. RUMGNA_02699 encodes a protein with homology with transcriptional regulators of the AraC family. The following 3 genes code for a predicted solute-binding protein (RUMGNA_02698) and two putative permeases (RUMGNA_02697, RUMGNA_02696), components of a sugar ABC transporter. The following gene has homology with oxidoreductase from the Gfo/Idh/MocA family. The sialidase gene nanH (RUMGNA_02694) predicted to encode the GH33 enzyme comes next. Then nanE (RUMGNA_02693), which encodes a predicted ManNAc-6-P epimerase is followed by nanA (RUMGNA_02692) encoding a putative Neu5Ac lyase. nanK (RUMGNA_02691) is the last gene of the cluster, coding for a predicted ManNAc kinase.

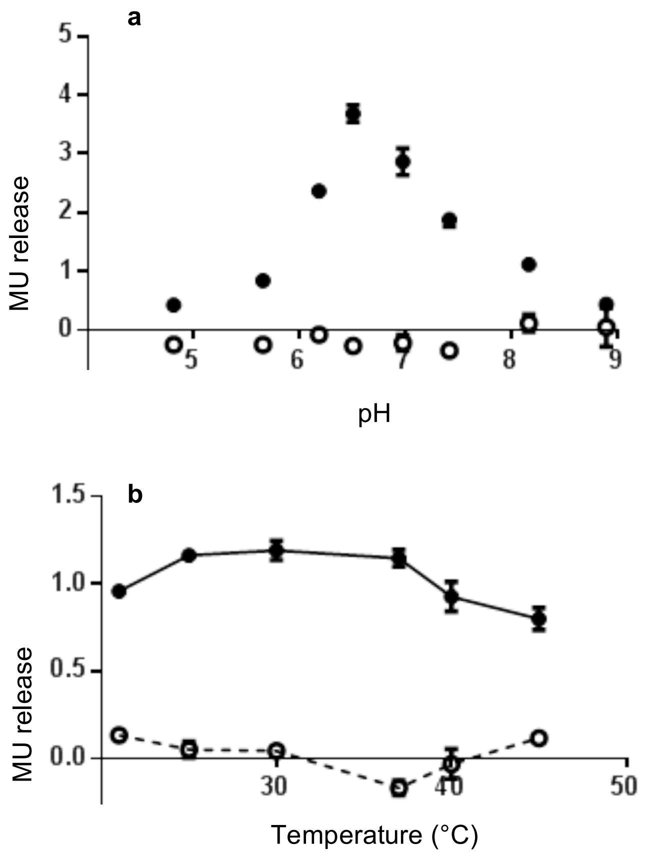

Supplementary Fig. 2 | pH and temperature-dependence of *Rg*NanH using 4MU-Neu5Ac as substrate

4MU-Neu5Ac (0.51 mM) was incubated in presence of 0.25 nM enzyme (filled circles) or in absence of enzyme (empty circles) as a control. The reaction was carried out in sodium phosphate buffer at different pHs (**a**) at 37°C, or at different temperatures (**b**) in phosphate buffered saline (PBS) at pH 7.4. The release of MU was measured by fluorimetry. The reaction was carried out in triplicate and the standard errors of the mean are shown.

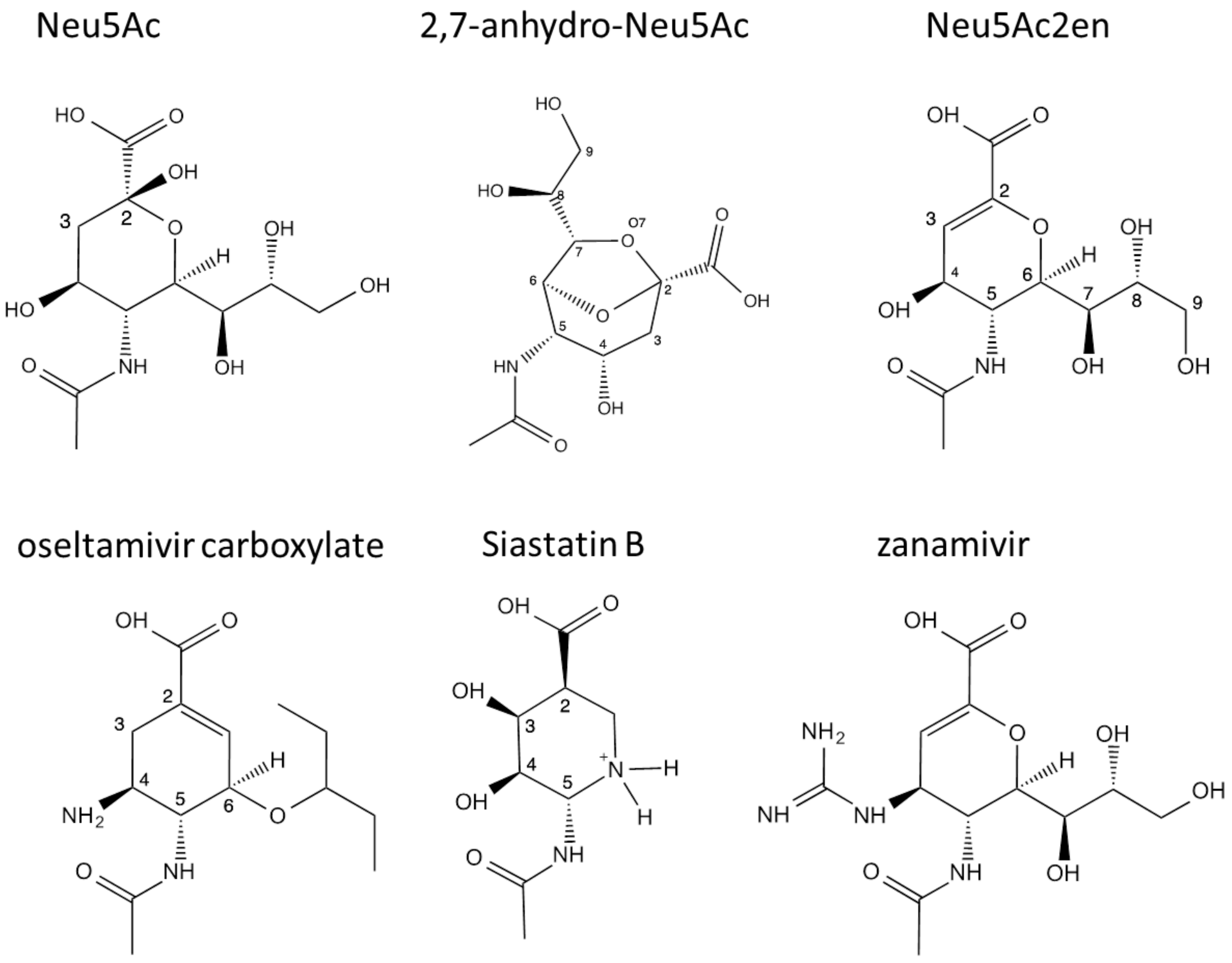

Supplementary Fig. 3 | Structures of inhibitors used in this study

The chemical structures of the inhibitors used in this study (Neu5Ac2en, oseltamivir carboxylate, Siastatin B, zanamivir) are shown here with Neu5Ac and 2,7-anhydro-Neu5Ac included for comparison. Atoms are numbered in relation to in text references.

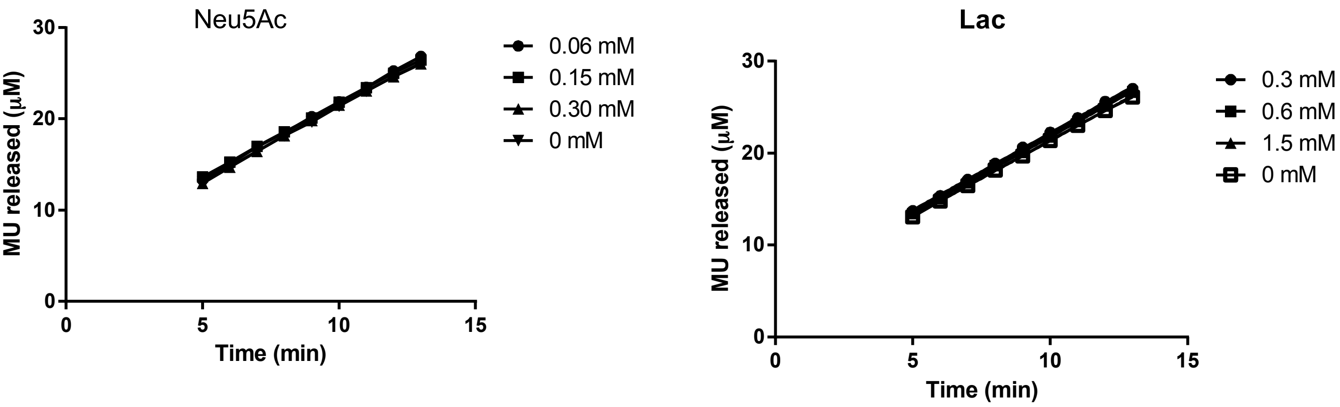

Supplementary Fig. 4 | Effect of Neu5Ac and Lac on *Rg*NanH

4MU-Neu5Ac (0.51 mM) was incubated in presence of 1 nM enzyme, with the indicated concentration of Neu5Ac (0-0.3 mM) or Lac (0-1.5 mM). The reaction was carried out in sodium phosphate buffer at pH 6.5 and at 37°C. The release of MU was measured by fluorimetry.

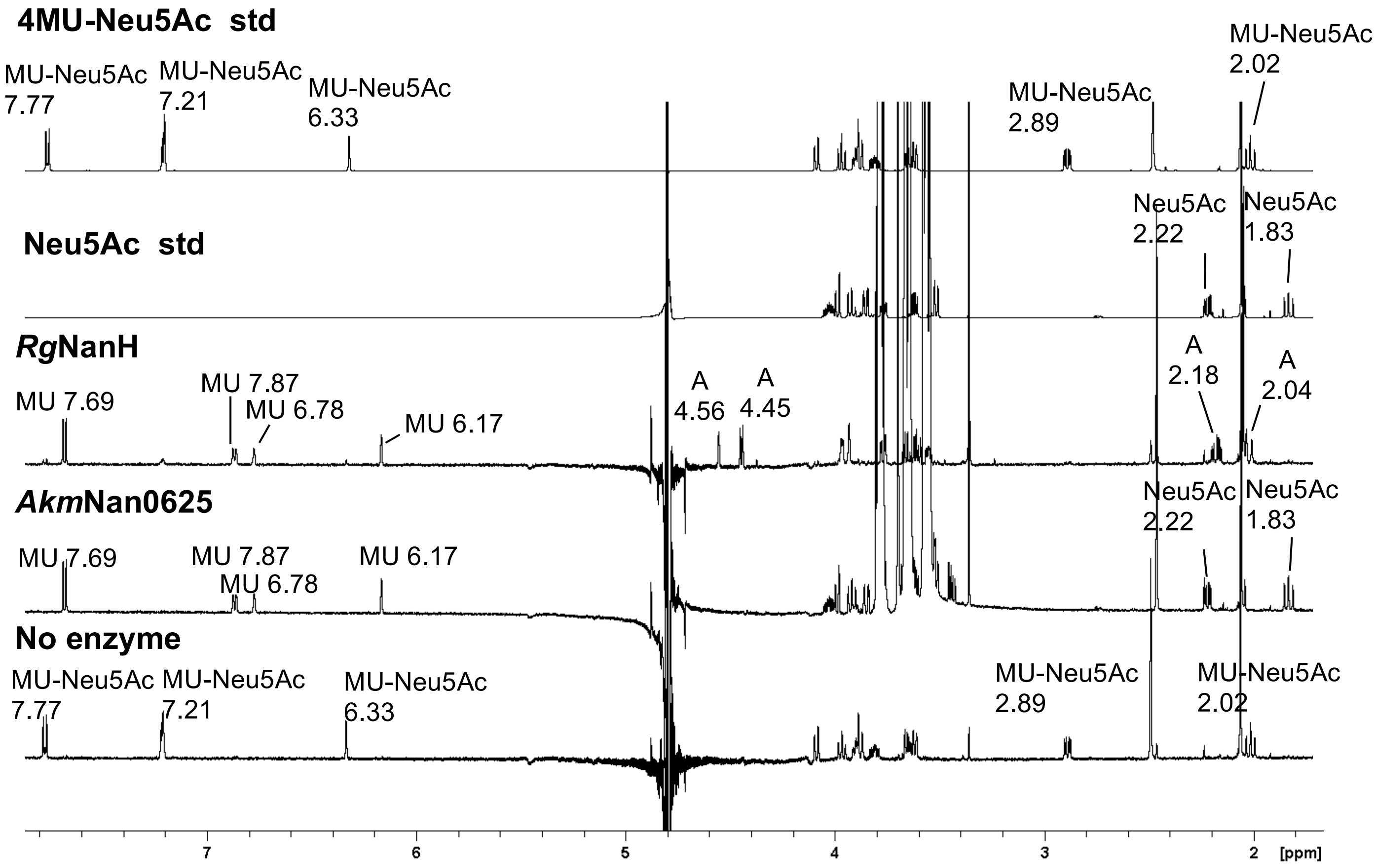

Supplementary Fig. 5 | Reaction products of *Rg*NanH and *Akm*Nan0625 enzymes incubated with 4MU-Neu5Ac

The sialidases were incubated with 4MU-Neu5Ac at 37°C, pH 6.5 overnight and the reaction products analysed by H^1 NMR. "A" corresponds to 2,7-anhydro-Neu5Ac. Spectra similar to *Akm*Nan0625 were obtained with *Akm*Nan1835 (not shown).

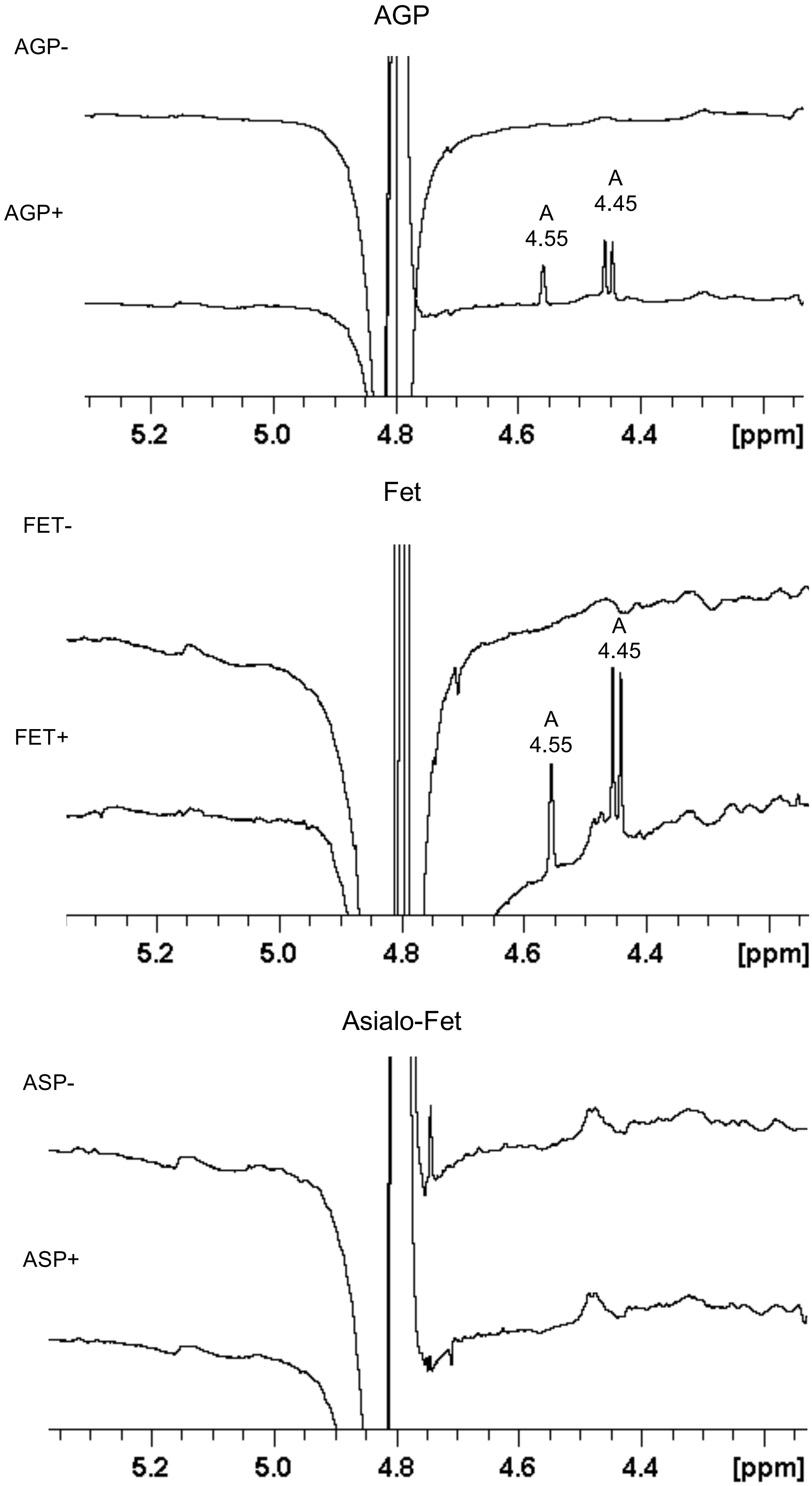
AGP
AGP-
AGP+
A
4.55
A
4.45
5.2
5.0
4.8
4.6
4.4
[ppm]
Fet
FET-
FET+
A
4.45
A
4.55
5.2
5.0
4.8
4.6
4.4
[ppm]
Asialo-Fet
ASP-
ASP+
5.2
5.0
4.8
4.6
4.4
[ppm]

Supplementary Fig. 6 | NMR Activity of *Rg*NanH against sialylated glycoproteins *Rg*NanH was incubated with human α-glycoprotein (AGP) and fetuin (Fet) (or asialo-fetuin as a control) at 37°C, pH 6.5 overnight and the reaction mixture analysed by H^1 NMR.

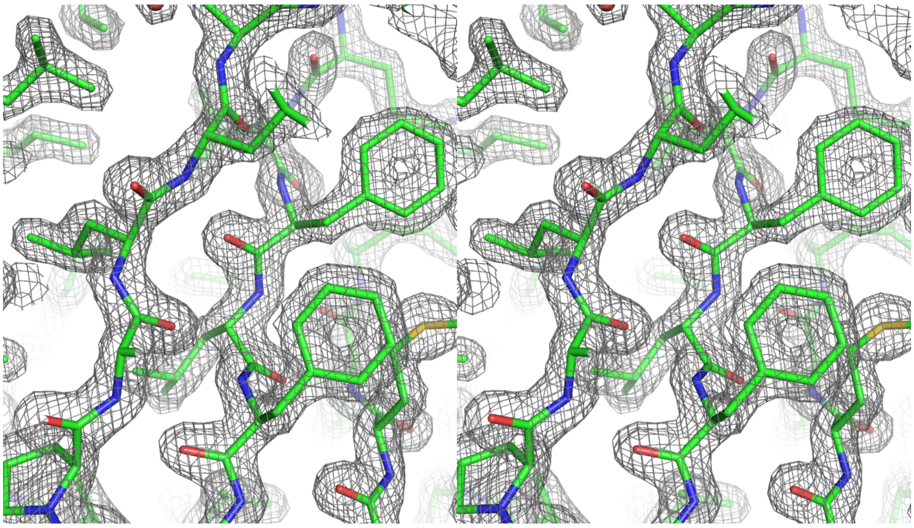

Supplementary Fig. 7| A wall-eye stereo image of a portion of the 2Fo-Fc electron density map of the *Rg*NanH 27-anhydro-Neu5Ac complex X-ray crystal structure. The map is contoured to 2.00 sigma.

Supplementary Table 1 | Signals of 2,7-anhydro-Neu5Ac and their chemical shifts

Proton chemical shifts for the non-exchangeable protons of 2,7-anhydro-Neu5Ac in D2O solution.

	Chemical shift (ppm)						
	H-3ax	H-3eq	H-4	H-5	H-6	H-7	5-Ac
H	2.18	2.02	4.09	3.94	4.56	4.45	2.05
C	35.44	35.44	67.85	52.16	77.01	76.63	21.93

Supplementary Table 2 | Occurrence of IT-sialidases and Nan clusters in genome-sequenced bacteria

Phylum	Class	Order	Family	Genus	Species	Number of genomes						
						Total[1]	with IT-sialidase	without IT-sialidase	with nan genes			without nan genes
									"classical" nan genes[2]		"*B. fragilis*-type" nan genes[4]	
									clustered[3]	not clustered		
**Firmicutes**	**Bacilli**	**Bacillales**	**Staphylo-coccaceae**	***Staphylococcus***	***S. pseudintermedius***	**2**	**2**	**0**	**0**	**2**	**0**	**0**
		**Lacto-bacillales**	**Lactobacillaceae**	***Lactobacillus***	***L. salivarius***	**7**	**3**	**4**	**6**	**1**	**0**	**0**
			**Streptococcaceae**	***Streptococcus***	***S. agalactiae***	**149**	**137**	**12**	**147**	**2**	**0**	**0**
					S. canis	**1**	**1**	**0**	**1**	**0**	**0**	**0**
					S. equi	**4**	**4**	**0**	**4**	**0**	**0**	**0**
					S. ictaluri	**1**	**1**	**0**	**1**	**0**	**0**	**0**
					S. infantis	**6**	**6**	**0**	**6**	**0**	**0**	**0**
					S. iniae	**2**	**2**	**0**	**2**	**0**	**0**	**0**
					S. intermedius	**6**	**5**	**1**	**6**	**0**	**0**	**0**
					S. mitis	**15**	**13**	**2**	**15**	**0**	**0**	**0**
					S. oralis	**10**	**10**	**0**	**10**	**0**	**0**	**0**
					S. peroris	**1**	**1**	**0**	**1**	**0**	**0**	**0**
					S. pneumoniae	**254**	**254**	**0**	**239**	**15**	**0**	**0**
					S. pseudo-pneumoniae	**3**	**3**	**0**	**3**	**0**	**0**	**0**
					S. sanguinis	**22**	**1**	**21**	**22**	**0**	**0**	**0**
					S. suis	**20**	**2**	**18**	**2**	**18**	**0**	**0**
					***S.* sp. BS35b**	**1**	**1**	**0**	**1**	**0**	**0**	**0**
					***S.* sp. C300**	**1**	**1**	**0**	**1**	**0**	**0**	**0**
					***S.* sp. F0441**	**1**	**1**	**0**	**1**	**0**	**0**	**0**
					***S.* sp. GMD1S**	**1**	**1**	**0**	**1**	**0**	**0**	**0**
					***S.* sp. GMD2S**	**1**	**1**	**0**	**0**	**0**	**0**	**1**
					***S.* sp. GMD4S**	**1**	**1**	**0**	**0**	**1**	**0**	**0**
					***S.* sp. GMD6S**	**1**	**1**	**0**	**0**	**1**	**0**	**0**

					***S.* sp. M143**	**1**	**1**	**0**	**1**	**0**	**0**	**0**
					***S.* sp. M334**	**1**	**1**	**0**	**1**	**0**	**0**	**0**
					***S.* sp. SK140**	**1**	**1**	**0**	**1**	**0**	**0**	**0**
					***S.* sp. SK643**	**1**	**1**	**0**	**1**	**0**	**0**	**0**
					***S.* sp. oral taxon 058**	**1**	**1**	**0**	**1**	**0**	**0**	**0**
					***S.* sp. oral taxon 071**	**1**	**1**	**0**	**1**	**0**	**0**	**0**
			**Carnobacteriaceae**	***Dolosigranulum***	***D. pigrum***	**1**	**1**	**0**	**1**	**0**	**0**	**0**
				Alloiococcus	***A. otitis***	**1**	**1**	**0**	**1**	**0**	**0**	**0**
	**Erysipelotrichia**	**Erysipelo-trichales**	**Erysipelo-trichaceae**	***Coprobacillus***	***C.* sp. 29_1**	**1**	**1**	**0**	**0**	**1**	**0**	**0**
				Erysipelato-clostridium	***Clostridium spiroforme*[5]**	**1**	**1**	**0**	**0**	**1**	**0**	**0**
	**Clostridia**	**Clostridiales**	**Lachnospiraceae**	***Blautia***	***B. hansenii***	**1**	**1**	**0**	**1**	**0**	**0**	**0**
					***Ruminococcus gnavus*[6,*]**	**5**	**4**	**1**	**4**	**1**	**0**	**0**
					***Ruminococcus torques*[6,**]**	**2**	**1**	**1**	**1**	**0**	**1**	**0**
				Tyzzerella	***Tyzzerella nexilis*[7]**	**1**	**1**	**0**	**0**	**0**	**0**	**1**
				**unclassified Lachnospiraceae**	**Lachnospiraceae bacterium 1_1_57FAA**	**1**	**1**	**0**	**0**	**0**	**1**	**0**
					**Lachnospiraceae bacterium 3_1_46FAA**	**1**	**1**	**0**	**0**	**0**	**1**	**0**
					**Lachnospiraceae bacterium 8_1_57FAA**	**1**	**1**	**0**	**0**	**0**	**1**	**0**
			**Clostridiaceae**	***Clostridium***	***C.* sp. 7_2_43FAA**	**1**	**1**	**0**	**1**	**0**	**0**	**0**
					C. celatum	**1**	**1**	**0**	**0**	**1**	**0**	**0**
					C. perfringens	**10**	**10**	**0**	**10**	**0**	**0**	**0**
**Proteobacteria**	**Gamma-proteobacteria**	**Pasteurellales**	**Pasteurellaceae**	***Pasteurella***	***P. multocida***	**11**	**8**	**3**	**9**	**2**	**0**	**0**

[1] Number of genomes with predicted protein-coding genes present in the NCBI Genomes database; a few strains of *S. infantis, S. oralis and S. pneumoniae* are represented by more than one genome.
[2] NanA, NanK, NanE(1) (*R. gnavus* nomenclature)
[3] the 3 nan genes are within a maximum of 15 consecutive genes.
[4] NanL, NanE(2), RokA (*B. fragilis* nomenclature)
[5] *Clostridium spiroforme* has been reclassified (Yutin and Galperin, 2013)
[6] *R. gnavus* and *R. torques* belong to a group of *Ruminococcus* spp. that have been reclassified in the genus *Blautia*, but have so far retained the *Ruminococcus* genus name (Liu et al, 2008).
[7] Formerly *Clostridium nexile* (Yutin and Galperin, 2013)

* One of the 5 genomes analyzed belongs to the E1 strain and is not publicly available. **One *R. torques* strain, which lacks the IT-sialidase, has the traditional NanA/K/E(1) cluster instead of the *B. fragilis* components.

Supplementary Methods

Bioinformatics Analysis. We searched all available genomes distributed by the NCBI (ftp://ftp.ncbi.nlm.nih.gov) for Nan clusters and "sialidase- encoding genes", as follows. We downloaded data (May 2014) for 2,771 complete (URI: /genomes/Bacteria) and 6,977 draft (/genomes/Bacteria_DRAFT) bacterial genomes, as well as 4,549 plasmid sequences (/genomes/Plasmids). These data included one *R. gnavus* strain (ATCC 29149) but we supplemented this with corresponding data from four other strains*:* CC55_001C and AGR2154 (both available at http://www.ncbi.nlm.nih.gov/genome/genomes/979 but omitted from the FTP distribution), E1[61] and ATCC 35913[62]. We used the provided annotations of protein-coding sequences and their products. A total of 8,238 genomes had available protein sequence data; these represented 8,126 unique strains of around 2,800 species. We searched all 28.6 million protein sequences for the presence of the domains of interest, and related these to the genomic locations of their corresponding genes for the purpose of determining gene clusters. Note that in cases where the sequence data are highly fragmented (due to genes occurring on different DNA fragment sequences, or occurring in missing genomic regions), the analysis of draft genomes may include some false negatives. We used profile Hidden Markov Models (pHMMS) of each protein domain as search queries, using HMMER3 version 3.1b1 (http://hmmer.org), using a maximum independent E-value of 10^{-4}. Where possible, we used available pHMMs from the Pfam database[63]; we verified their suitability by comparing performance on test data (not shown) with pHMMs constructed from our own alignments of homologues of *R. gnavus* sequences. This confirmed that Pfam domains (i) PF00701 ("DHDPS"), (ii) PF00480 ("ROK"), (iii) PF04131 ("NanE"), and (iv) PF07221 ("GlcNAc_2-epim") were very suitable models respectively for (i) *R. gnavus* NanA (encoded by RUMGNA_02692) and homologues (including *B. fragilis* NanL); (ii) *R. gnavus* NanK (RUMGNA_02691) and homologues; (iii) *R. gnavus* NanE (RUMGNA_02691) and homologues (including *E. coli* NanE[14]); (iv) *B. fragilis* NanE[16] and homologues (this NanE is an epimerase homologous to renin-binding proteins). For clarity, we refer to these four domains as NanA, NanK, NanE1, NanE2. For NanH and homologues, we built our own model from an alignment of *Rg*NanH (RUMGNA_02694) and 984 sequences from the GH33 family (www.cazy.org); this alignment was edited to remove N- and C-terminal segments which are absent from *Rg*NanH, as well as a segment corresponding to the I-domain. This model (which we refer to as "Sialidase") therefore detected both conventional GH33 domains as a contiguous match, and also *Rg*NanH-type domains as a segmented match with a

gap representing the location of the I-domain. We built a model of the I-domain itself from the corresponding segments in the CAZy GH33 sequences (132 sequence segments). Note that the Pfam domain PF02973 named "Sialidase" appears to correspond to the carbohydrate-binding module CBM40 (www.cazy.org), so we did not use this to search for NanH. However, we built our own model named "CBM40", from 36 sequence segments from the CAZy database, corresponding to the *Rg*NanH CBM40 domain; this performed similarly to PF02973. Gene clusters: we defined clusters as N consecutive gene loci where N is the smallest number such that all genes of interest are present in any order, and $N \leq N_{max}$, the largest permitted cluster size. We found that the number of genomes positive for a cluster of NanA, NanK, and NanE1 effectively plateaued at $N_{max} = 15$, so we used that as the maximum size (87% of all genomes with these three genes on the same genomic sequence fulfil $N \leq 15$). Human metagenome data: we translated the assembled coding sequences of the metagenomes of the 125 subjects (99 with no IBD, 26 with IBD) described in Table S1 of reference[10] (MetaHIT Consortium study) prior to searching with HMMER3 as before. We found this method to be much more sensitive than BLASTP (or TBLASTN on the coding sequences themselves) searches even with a liberal BLAST E-value cut-off of 10^{-3}. Manual inspection of a sample of the additional hits arising from HMMER3 compared to BLAST indicated that they were clearly homologous to the query sequences. Analyses and post-processing were automated by Perl scripts, including some BioPerl modules (Stajich J.E. *et al.* (2002), *Genome Res.* **12**(10) 1611-8).
